# Supplementary material for: Transcriptomic Analyses of Grapevine Leafroll-Associated Virus 3 Infection in Leaves and Berries of ‘Cabernet Franc’
Source: Viruses. 2022 Aug 21;14(8):1831. doi: 10.3390/v14081831 (PMC9415066; doi:10.3390/v14081831)
Supplement: Supplementary file 1 [file viruses-14-01831-s001.zip › Song et al. RNA-Seq grapevine-GLRaV-3 STable S6 Key biological process and its associated genes.pdf]

Table S6 Key biological process and its associated genes differentially regulated in GLRaV-3-infected leaf and berry at E-L 31, 35, and 38 developmental stages.

**Degree of expressional change (fold change) and significant difference (FDR values) were calculated by differential expression analysis with DESeq2. For full list of differentially expressed genes (DEGs) and their associated fold change and FDR values at each developmental stages of leaf and berry, please refer to STable 4.*

**The genes included are DEGs that past FDR <0.05 threshold.*
**Gene ID and gene description were retrieved from Ensembl plants.*
**N/A indicates that no DEG was detected at FDR <0.05 threshold.*

A. Down-regulation

Cellular process	Tissue	Developmental stage	Gene ID	Gene description	Fold change	FDR value
Photosynthesis	Leaf	E-L 31	VIT_08s0040g00850	PSBP domain-containing protein 6, chloroplastic	-1.99199599	0.033903241
			VIT_14s0128g00030	PSBP domain-containing protein	-1.650587644	0.017176662
			VIT_00s0181g00180	Chlorophyll a-b binding protein 13, chloroplastic	-1.431019538	0.003692198
			VIT_13s0019g00260	Putative oxygen evolving enhancer protein	-1.308234815	0.006368829
			VIT_13s0019g00320	Photosynthetic NDH subunit of lumenal location 1, chloroplastic	-1.2937063	0.020103386
			VIT_19s0014g05080	Uncharacterized protein	-1.292630128	0.000480496
			VIT_14s0081g00060	PSII 6.1 kDa protein	-1.211161416	0.002141469
			VIT_10s0003g02900	Chlorophyll a-b binding protein of LHCII type 1	-1.185162996	0.001994627

			VIT_00s0904g00010	Oxygen-evolving enhancer protein 3, chloroplastic	-1.168540939	0.002847964
			VIT_10s0116g00900	Chaperonin-like RBCX protein 1, chloroplastic	-1.063521336	0.029491686
			VIT_19s0015g01760	PSI-G	-0.829651289	0.04323668
			VIT_10s0003g04350	PSI reaction center subunit psaK, chloroplastic	-0.828464574	0.020685447
			VIT_11s0016g01400	Uncharacterized protein	-0.780210214	0.020463039
			VIT_12s0028g00320	Chlorophyll a-b binding protein 40, chloroplastic-like	-0.773527941	0.023554993
	Leaf	E-L 35	*A total of 49 down-regulated DEGs were involved in photosynthesis, for detailed list of down-DEGs involved in photosynthesis, please refer to S Table 5, 'E-L 35 Leaf' subsheet			
	Berry	E-L 31	N/A	N/A	N/A	N/A
	Berry	E-L 35	N/A	N/A	N/A	N/A
	Berry	E-L 38	N/A	N/A	N/A	N/A
Chloroplast rRNA processing	Leaf	E-L 31	VIT_13s0064g01430	30S ribosomal protein 2, chloroplastic	-1.274681552	0.016161032
			VIT_17s0000g09680	28 kDa ribonucleoprotein, chloroplastic	-1.161692835	0.005982332
			VIT_14s0066g00140	28 kDa ribonucleoprot	-0.874158366	0.017212347

				ein, chloroplastic		
			VIT_08s0040g01760	29 kDa ribonucleoprotein A, chloroplastic	-0.866751862	0.01161672
	Leaf	E-L 35	N/A	N/A	N/A	N/A
	Berry	E-L 31	N/A	N/A	N/A	N/A
	Berry	E-L 35	N/A	N/A	N/A	N/A
	Berry	E-L 38	N/A	N/A	N/A	N/A

Plastid transcription	Leaf	E-L 31	VIT_01s0011g01080	Protein PLASTID TRANSCRIPTIONALLY ACTIVE 14	-1.312743353	0.044436754
			VIT_01s0011g00240	Fructokinase-like 2, chloroplastic	-1.116663713	0.029280554
			VIT_07s0104g00720	Transcription termination factor MTERF4, chloroplastic	-0.918594687	0.022533095
	Leaf	E-L 35	N/A	N/A	N/A	N/A
	Berry	E-L 31	N/A	N/A	N/A	N/A
	Berry	E-L 35	N/A	N/A	N/A	N/A
	Berry	E-L 38	N/A	N/A	N/A	N/A

Chlorophyll biosynthetic process	Leaf	E-L 31	N/A	N/A	N/A	N/A
	Leaf	E-L 35	VIT_08s0007g08540	CHLH magnesium	-2.698812022	0.00061374

				chelatase H subunit		
			VIT_19s0014g03160	Protochlorophyllide reductase, chloroplastic	-2.655802771	1.35202E-06
			VIT_06s0004g08380	Uncharacterized protein	-2.111207648	0.001493512
			VIT_08s0040g00390	Magnesium-protoporphyrin IX monomethyl ester [oxidative] cyclase, chloroplastic-like	-2.064978918	0.006833531
			VIT_12s0059g00270	Protochlorophyllide reductase	-1.918082208	0.004501822
			VIT_05s0020g02130	Probable 1-deoxy-D-xylulose-5-phosphate synthase, chloroplastic	-1.627046679	0.006092146
			VIT_13s0067g02450	Uroporphyrinogen decarboxylase	-1.341399694	0.037902476
	Berry	E-L 31	N/A	N/A	N/A	N/A
	Berry	E-L 35	N/A	N/A	N/A	N/A
	Berry	E-L 38	N/A	N/A	N/A	N/A

Carbohydrate metabolism	Leaf	E-L 31	VIT_11s0016g00470	Sucrose synthase (VvSUS)	-1.548123753	0.006430004
			VIT_05s0077g00510	Neutral invertase (VvNIV)	-1.308977202	0.008078829

	Leaf	E-L 35	VIT_12s0059g02670	Beta-amylase (VvBMY)	-2.308800198	0.042186627
			VIT_05s0020g01910	Beta-amylase (VvBMY)	-1.961451369	0.000320976
			VIT_08s0007g01570	Fructose bisphosphates (VvFBP)	-1.839839925	0.011699588
			VIT_05s0077g00510	Neutral invertase (VvNIV)	-1.764111238	0.005289888
			VIT_02s0109g00080	Phosphoribulokinase (VvPRK)	-1.759907347	0.012733449
			VIT_03s0038g00380	D-ribulose kinase (VvPSK)	-1.71631345	0.040854439
			VIT_02s0154g00100	Transaldolase (VvTALDO)	-1.625053341	0.011384389
			VIT_01s0011g05210	Phosphoglucomutase (VvPGM)	-1.453673213	0.010979642
			VIT_17s0000g02260	Aldose 1-epimerase	-1.287142274	0.049904321
	Berry	E-L 31	VIT_00s2840g00010	Phosphoenolpyruvate carboxykinase (VvPEPCK)	-0.526783839	0.043710289
			VIT_00s2576g00010	Phosphoenolpyruvate carboxykinase (VvPEPCK)	-0.44549967	0.042861108
			VIT_00s1995g00010	Phosphoenolpyruvate carboxykinase (VvPEPCK)	-0.426019813	0.012855684
	Berry	E-L 35	N/A	N/A	N/A	N/A
	Berry	E-L 38	N/A	N/A	N/A	N/A

Cell wall metabolism	Leaf	E-L 31	VIT_01s0026g02620	Expansin	-4.110101857	0.011963935

			VIT_14s0108g01020	Expansin	-3.80167402	3.20E-06
			VIT_13s0067g02930	Expansin	-3.403329287	0.046829039
			VIT_02s0025g01330	Polygalacturonase	-1.168541952	0.00578332
	Leaf	E-L 35	VIT_13s0064g01720	Beta-galactosidase	-9.415734344	0.000436275
			VIT_13s0067g02930	Expansin	-7.293236121	1.0373E-06
			VIT_06s0004g07970	Expansin	-7.023100639	0.032065526
			VIT_07s0031g02480	Beta-galactosidase	-5.269973299	0.000630032
			VIT_02s0025g01330	Polygalacturonase	-2.056273929	0.01842931
			VIT_09s0002g02120	Beta-galactosidase	-1.883018002	0.014438646
	Berry	E-L 31	N/A	N/A	N/A	N/A
	Berry	E-L 35	N/A	N/A	N/A	N/A
	Berry	E-L 38	N/A	N/A	N/A	N/A

Sugar transport	Leaf	E-L 31	N/A	N/A	N/A	N/A
	Leaf	E-L 35	VIT_18s0076g00220	Sucrose transporter 2 (VvSUT2)	-8.189513873	0.026456582
			VIT_04s0023g01500	Putative polyol/monosaccharide transporter (VvPMT3)	-3.344359148	0.000909145
			VIT_18s0076g00250	Sucrose transporter 27 (VvSUT27)	-2.750551874	9.13414E-06
			VIT_11s0149g00050	Hexose transporter 3/7 (VvHT3/7)	-2.030396829	0.005609524
	Berry	E-L 31	N/A	N/A	N/A	N/A

	Berry	E-L 35	N/A	N/A	N/A	N/A
	Berry	E-L 38	N/A	N/A	N/A	N/A

Mitochondrion-associated	Leaf	E-L 31	N/A	N/A	N/A	N/A
	Leaf	E-L 35	VIT_16s0039g02750	Glutamate dehydrogenase (VvGDH)	-2.553537017	0.040452982
			VIT_00s1286g00020	Translation elongation factor	-2.197594459	0.001509423
			VIT_09s0002g04330	Mitochondrial ribosomal protein	-1.965057526	0.003534502
			VIT_19s0015g00140	Mitochondrial ribosomal protein	-1.917158837	0.005272773
			VIT_18s0001g03850	Mitochondrial ribosomal protein	-1.706629018	0.006407113
			VIT_00s0323g00080	Translation elongation factor	-1.681144532	0.008073655
			VIT_05s0049g00470	Electron transfer flavoprotein subunit beta	-1.40078102	0.037958832
			VIT_19s0014g04970	Mitochondrial ribosomal protein	-1.33398672	0.049830236
	Berry	E-L 31	N/A	N/A	N/A	N/A
	Berry	E-L 35	VIT_06s0009g03590	Reactive oxygen species modulator 1 (VvROMO1)	-1.034816368	0.038007393
			VIT_05s0020g02150	NADH dehydrogenase [ubiquinone] 1 alpha	-0.97007366	0.015243494

				subcomplex subunit 8-B		
			VIT_01s0137g00340	Uncharacterized protein	-0.959519843	0.032710584
			VIT_02s0025g02990	Mitochondrial import inner membrane translocase subunit TIM13 (VvTIM13)	-0.955229294	0.033015689
			VIT_11s0103g00270	NADH dehydrogenase [ubiquinone] 1 alpha subcomplex subunit 1	-0.916141105	0.033455189
			VIT_06s0080g00540	Uncharacterized protein	-0.783549942	0.0398873
	Berry	E-L 38	N/A	N/A	N/A	N/A

Phenylpropanoids and flavonoids biosynthesis	Leaf	E-L 31	N/A	N/A	N/A	N/A
	Leaf	E-L 35	N/A	N/A	N/A	N/A
	Berry	E-L 31	VIT_02s0033g00260	Isoflavone reductase-like protein 1 (VvIFRL1)	-1.109011296	0.034272172
			VIT_06s0004g06390	UDP-glycosyltransferase 71K1 (VvUGT71K1)	-0.973356038	4.25304E-07
	Berry	E-L 35	VIT_00s0361g00040	Anthocyanidin reductase (VvANR)	-1.398423406	0.001855379
			VIT_01s0011g02960	Leucoanthocyanidin reductase 1 (VvLAR1)	-0.858890334	0.040345807

	Berry	E-L 38	N/A	N/A	N/A	N/A

Defense mechanism	Leaf	E-L 31	N/A	N/A	N/A	N/A
	Leaf	E-L 35	VIT_05s0077g01670	Pathogenesis-related protein 10.7 (VvPR-10.7)	-9.930153917	6.44732E-05
	Berry	E-L 31	N/A	N/A	N/A	N/A
	Berry	E-L 35	N/A	N/A	N/A	N/A
	Berry	E-L 38	VIT_05s0077g01670	Pathogenesis-related protein 10.7 (VvPR-10.7)	-1.134068074	0.000626765

Heat shock protein (HSP) and HSP co-chaperons	Leaf	E-L 31	N/A	N/A	N/A	N/A
	Leaf	E-L 35	VIT_15s0046g01560	BAG family molecular chaperone regulator 6 (BAG6)	-2.962246735	2.19805E-07
			VIT_18s0001g01570	16.9kDa HSP class I	-2.703856226	0.00012535
			VIT_02s0025g00280	HSP83	-1.967049793	0.016661365
			VIT_15s0021g00830	Chaperonin GroES	-1.704049014	0.006114618
			VIT_09s0002g03930	HSP60	-1.670062319	0.036612176
	Berry	E-L 31	N/A	N/A	N/A	N/A
	Berry	E-L 35	N/A	N/A	N/A	N/A
	Berry	E-L 38	N/A	N/A	N/A	N/A

Transcription factors	Leaf	E-L 31	N/A	N/A	N/A	N/A
	Leaf	E-L 35	VIT_00s0226g00070	Protein SHORT ROOT/SHR	-10.74683952	1.69584E-07
			VIT_14s0108g00420	Transcription factor PRE2	-8.138166707	0.029248163
			VIT_04s0008g05560	IAA28	-7.66134994	6.61887E-06
			VIT_08s0056g00050	Protein SCARECROW/SCR	-6.384202169	0.023163259
			VIT_05s0020g04690	IAA14	-2.519923737	3.06575E-05
			VIT_15s0046g00290	ARF18	-1.982160394	0.002477448
			VIT_11s0016g03540	IAA27	-1.51840453	0.012627664
			VIT_02s0025g01740	ARF9	-1.333772382	0.037833687
			VIT_18s0001g08090	IAA	-1.296871905	0.024004196
	Berry	E-L 31	N/A	N/A	N/A	N/A
	Berry	E-L 35	VIT_11s0016g00660	ERF017	-5.759829656	0.001535734
			VIT_03s0063g00460	ERF109	-3.959097156	0.005743998
			VIT_16s0013g00950	ERF105	-3.939262956	0.024882223
			VIT_16s0013g01110	ERF005	-3.268995095	0.020280177
			VIT_16s0013g00990	ERF005	-2.643451912	0.044893097
			VIT_07s0141g00690	ERF011	-2.337779483	0.009044125
			VIT_01s0011g03070	ERF RAV1	-2.326366551	0.0453749
			VIT_12s0028g03270	ERF004	-2.144067757	0.011025863
			VIT_19s0014g02240	ERF004	-1.853191933	0.013494055
			VIT_18s0089g01030	ERF TINY	-1.755868123	0.003373735

			VIT_10s0003g00140	ERF004	-1.589187054	0.012139086
			VIT_07s0031g00190	ERF008	-1.363387699	0.011403663
			VIT_02s0234g00130	ERF002	-1.284901335	0.026560263
	Berry	E-L 38	N/A	N/A	N/A	N/A

B. Up-regulation

Cellular process	Tissue	Developmental stage	Gene ID	Gene description	Fold change	FDR value
Photosynthesis	Leaf	E-L 31	N/A	N/A	N/A	N/A
	Leaf	E-L 35	N/A	N/A	N/A	N/A
	Berry	E-L 31	VIT_00s0181g00180	Chlorophyll a-b binding protein, chloroplastic	0.978761274	2.58407E-05
			VIT_08s0007g08540	Magnesium chelatase	0.948702186	0.001118541
			VIT_12s0028g00320	Chlorophyll a-b binding protein, chloroplastic	0.843803366	0.029337049
			VIT_19s0014g00160	Chlorophyll a-b binding protein, chloroplastic	0.830805631	0.024482006
			VIT_18s0001g10550	Chlorophyll a-b binding protein, chloroplastic	0.684770452	0.022418583
			VIT_04s0044g01410	Uncharacterized protein	0.682304447	0.000698058
			VIT_07s0005g02220	Chlorophyll a-b binding protein, chloroplastic	0.625285518	0.000100081

			VIT_12s0055g01110	Chlorophyll a-b binding protein, chloroplastic	0.6148984	0.008966202
			VIT_04s0008g01730	Uncharacterized protein	0.573757864	0.04184377
			VIT_10s0003g04350	PSI-K (Photosystem I subunit X)	0.568360169	0.045472904
			VIT_17s0000g03690	Ribulose bisphosphate carboxylase small subunit (RuBisCO small subunit)	0.561154296	0.001145619
			VIT_08s0040g00390	Magnesium-protoporphyrin IX monomethyl ester (oxidative) cyclase	0.559713019	0.002928141
			VIT_07s0005g04400	Photosystem II reaction center Psb28 protein	0.536245052	0.021864576
			VIT_10s0003g02900	Chlorophyll a-b binding protein, chloroplastic	0.52523484	0.000740937
			VIT_19s0014g03850	Plastoquinol--plastocyanin reductase	0.500333908	0.012590557
			VIT_00s0207g00210	Uncharacterized protein	0.487967068	0.008653088
			VIT_12s0057g00020	Uncharacterized protein	0.459643508	0.002755571
			VIT_00s0904g00010	Uncharacterized protein	0.453728805	0.004595422
			VIT_15s0024g00040	Chlorophyll a-b binding protein, chloroplastic	0.448962562	0.043278122
			VIT_04s0023g00410	PSI subunit V	0.445005561	0.026789559
	Berry	E-L 35	N/A	N/A	N/A	N/A
	Berry	E-L 38	N/A	N/A	N/A	N/A

Carbohydrate metabolism	Leaf	E-L 31	N/A	N/A	N/A	N/A
	Leaf	E-L 35	VIT_02s0025g02790	Granule-bound starch synthase 1, chloroplastic/amyloplastic (VvGBSS1)	2.432168517	0.029050767
			VIT_18s0001g00560	Alpha-amylase (VvAMY)	2.107962246	0.03403715
			VIT_03s0063g00400	Alpha-amylase (VvAMY)	1.865813776	0.002551314
	Berry	E-L 31	VIT_03s0038g00670	Aldolase and fructose bisphosphatase (VvFBP)	1.127195337	0.000300258
			VIT_08s0007g01570	Aldolase and fructose bisphosphatase (VvFBP)	1.041426285	8.76918E-06
			VIT_04s0023g03010	Aldolase and fructose bisphosphatase (VvFBP)	0.730823016	0.006163961
			VIT_13s0019g03350	Aldolase and fructose bisphosphatase (VvFBP)	0.67705116	0.003234988
			VIT_17s0000g03690	RuBisCO small subunit	0.561154296	0.001145619
			VIT_12s0028g02180	PEP carboxylase (VvPEPCase)	0.409252683	0.007891183
			VIT_01s0011g02740	PEP carboxylase (VvPEPCase)	0.387905072	0.021337078
	Berry	E-L 35	VIT_05s0077g01930	Sucrose synthase (VvSUS)	3.569665361	0.015715931
	Berry	E-L 38	VIT_11s0016g00470	Sucrose synthase (VvSUS)	0.980264567	6.03957E-06

Cell wall metabolism	Leaf	E-L 31	VIT_01s0026g00200	Xyloglucan endotransglucosylase/hydrolase	5.681804968	0.002130956

		VIT_02s0025g01890	Cellulose synthase	4.803366519	0.004353587
		VIT_02s0025g01920	Cellulose synthase	3.98557283	0.049377963
		VIT_02s0025g01750	Cellulose synthase	1.550531121	0.007177193
		VIT_05s0049g00010	Cellulose synthase	1.252825821	0.013575809
		VIT_19s0015g00730	Cellulose synthase	1.21844233	2.78E-05
		VIT_05s0020g05050	Cellulose synthase	1.155965674	0.013262747
Leaf	E-L 35	VIT_05s0094g00330	Chitinase-related protein	10.65846019	3.54211E-05
		VIT_01s0026g00200	Xyloglucan endotransglucosylase/hydrolase	5.374521331	2.46673E-05
		VIT_12s0134g00160	Xyloglucan endotransglucosylase/hydrolase	5.321779547	0.003767351
		VIT_11s0052g01200	Xyloglucan endotransglucosylase/hydrolase	4.712408319	1.26785E-05
		VIT_15s0046g01570	Endochitinase	2.461538849	0.00565505
		VIT_04s0008g00140	Chitinase-related protein	2.310004982	0.000200818
Berry	E-L 31	VIT_15s0048g00510	Pectinesterase (VvPE)	2.316820998	0.049579898
		VIT_06s0004g06210	Cytochrome P450 86A1 (VvCYP86A1)	1.445183446	0.027594962
		VIT_09s0002g08510	Expansion	1.194644722	0.013455533
		VIT_05s0062g00240	Xyloglucan endotransglucosylase/hydrolase	0.922226533	0.000834196
		VIT_08s0007g04950	Xyloglucan endotransglucosylase/hydrolase	0.921287429	0.018337571
		VIT_11s0052g01250	Xyloglucan endotransglucosylase/hydrolase	0.858683769	0.009814869
		VIT_11s0016g00330	Pectinesterase (VvPE)	0.761027332	0.000698058

			VIT_19s0014g00450	Protein trichome birefringence	0.738553035	0.005976317
			VIT_14s0083g01100	UDP-arabinopyranose mutase 3	0.703600045	0.002134208
			VIT_14s0108g01020	Expansion	0.667604626	0.000278588
			VIT_08s0007g03990	Glucomannan 4-beta-mannosyltransferase	0.624859159	0.014892072
			VIT_05s0029g00480	Eceriferum 26	0.472821932	0.007634942
			VIT_17s0000g05070	COBRA	0.44238972	0.015022359
	Berry	E-L 35	N/A	N/A	N/A	N/A
	Berry	E-L 38	N/A	N/A	N/A	N/A

Sugar transport	Leaf	E-L 31	N/A	N/A	N/A	N/A
	Leaf	E-L 35	VIT_18s0001g05570	Hexose transporter 2 (VvHT2)	2.619411857	0.000297238
			VIT_01s0026g01890	UDP-galactose transporter 3-like (VvUTR3-like)	1.982264626	0.005963856
			VIT_05s0020g03140	Hexose transporter 5 (VvHT5)	1.708355616	0.001692151
	Berry	E-L 31	VIT_03s0063g02250	Polyol transporter 5 (VvPLT5)	0.961300985	0.021337078
			VIT_10s0003g03930	Inositol transporter 2 (VvINT2)	0.580244901	0.006415565
			VIT_05s0077g02260	Bidirectional sugar transporter SWEET17 (VvSWEET17)	0.464126135	0.020862053
			VIT_10s0003g00300	Triose phosphate/phosp	0.389103626	0.015022359

				hate translocator (VvTPT)		
	Berry	E-L 35	N/A	N/A	N/A	N/A
	Berry	E-L 38	VIT_18s0076g00250	Sucrose transporter 27 (VvSUT27)	1.027053423	0.025598535
Phenylpropanoids and flavonoids biosynthesis	Leaf	E-L 31	N/A	N/A	N/A	N/A
	Leaf	E-L 35	VIT_01s0011g02960	Leucoanthocyanidin reductase 1 (VvLAR1)	10.48107095	0.032371647
			VIT_16s0039g02230	Anthocyanidin 3-O-glucosyltransferase (VvUFGT)	9.370984519	0.018772542
			VIT_02s0025g04720	Anthocyanidin synthase (VvANS)	7.826568924	0.00109728
			VIT_04s0023g03370	Flavonol synthase/flavanone 3-hydroxylase (VvFLS)	6.255754492	0.032054866
			VIT_18s0001g12800	Dihydroflavonol 4-reductase (VvDFR)	4.152928318	0.000128398
			VIT_18s0001g03430	Flavonol synthase/flavanone 3-hydroxylase (VvFLS)	3.46402729	0.024144198
			VIT_00s0361g00040	Anthocyanidin reductase (VvANR)	2.949824018	0.030812789
	Berry	E-L 31	N/A	N/A	N/A	N/A
	Berry	E-L 35	N/A	N/A	N/A	N/A
	Berry	E-L 38	VIT_00s0615g00020	Mannitol dehydrogenase (VvCAD7)	2.897829319	0.000385188

	VIT_16s0039g01360	Phenylalanine ammonia lyase (VvPAL)	2.684839342	0.043559059
	VIT_16s0039g01170	Phenylalanine ammonia lyase (VvPAL)	2.52034501	5.17849E-07
	VIT_00s0615g00030	Mannitol dehydrogenase (VvCAD7)	2.382416367	0.002517939
	VIT_16s0039g01300	Phenylalanine ammonia lyase (VvPAL)	2.353836295	0.001025274
	VIT_16s0039g01110	Phenylalanine ammonia lyase (VvPAL)	2.265560934	0.022309729
	VIT_16s0100g01000	Stilbene synthase 4-like (VvSTS4-like)	2.022234337	0.002041952
	VIT_16s0100g00850	Stilbene synthase 4-like (VvSTS4-like)	1.989697388	0.030874368
	VIT_12s0034g00130	Anthocyanidin 3-O-glucosyltransferase 2 (VvUFGT)	1.842701647	0.001464303
	VIT_04s0044g00210	Mannitol dehydrogenase (VvCAD7)	1.212897654	0.026116134
	VIT_06s0004g08150	Trans-cinnamate 4-monooxygenase (VvC4H)	1.071779028	0.01764136
	VIT_07s0031g00350	Caffeoyl-CoA O-methyltransferase (VvCCOAMT)	1.00716124	0.000626765
	VIT_17s0000g04150	Leucoanthocyanidin reductase 2 (VvLAR2)	0.920483688	0.048838407
	VIT_08s0040g03040	Glutathione S-transferase L3 (VvGSTL3)	0.748200655	0.006085753

Defense mechanism	Leaf	E-L 31	VIT_06s0061g00100	Beta-1,3-glucanase (VvPR-2)	4.595860638	0.033320268
			VIT_05s0077g01550	Pathogenesis-related protein 10.3 (VvPR-10.3)	1.338843305	0.035579663
	Leaf	E-L 35	VIT_02s0025g04300	Thaumatin-like protein (VvPR-5)	7.131637772	0.04494005
			VIT_02s0025g04290	Thaumatin-like protein (VvPR-5)	6.396709659	4.16405E-05
			VIT_06s0061g00120	Beta-1,3-glucanase (VvPR-2)	3.938911873	0.001383384
			VIT_00s0184g00120	Toll/interleukin-1 receptor/resistance (TIR) domain-containing protein	3.885911564	0.018465883
			VIT_02s0025g04230	Thaumatin-like protein (VvPR-5)	3.283661665	0.020321187
			VIT_14s0081g00030	Pathogenesis-related protein 4A (VvPR-4A)	3.181712348	0.021084037
			VIT_02s0025g04250	Thaumatin-like protein (VvPR-5)	3.148840994	0.009186248
			VIT_18s0001g03710	Toll/interleukin-1 receptor/resistance (TIR) domain-containing protein	2.895183845	0.003391152
			VIT_05s0029g00880	NB-ARC domain-containing protein	2.511487926	0.035542064
			VIT_05s0077g01570	Pathogenesis-related protein 10.3 (VvPR-10.3)	2.143373036	0.024144198
			VIT_05s0077g01550	Pathogenesis-related protein 10.3 (VvPR-10.3)	1.633264	0.015467

		VIT_17s0000g07400	protein EDS1-related	1.632824173	0.036783159
		VIT_17s0000g07560	protein EDS1-related	1.368062009	0.04525429
Berry	E-L 31	N/A	N/A	N/A	N/A
Berry	E-L 35	VIT_13s0047g00950	RN_N domain-containing protein	2.753895328	0.039394598
		VIT_05s0077g01590	Pathogenesis-related protein 10.3 (VvPR-10.3)	2.490038546	0.040953162
		VIT_00s0467g00010	RN_N domain-containing protein	2.47789475	0.016759386
		VIT_13s0101g00300	RN_N domain-containing protein	2.414197528	0.025370541
		VIT_13s0158g00210	RN_N domain-containing protein	2.303466838	0.01256742
		VIT_13s0158g00260	RN_N domain-containing protein	2.097576895	0.03720172
		VIT_07s0141g01030	RN_N domain-containing protein	2.071084294	0.018534408
		VIT_12s0034g01490	RN_N domain-containing protein	1.975898303	0.019977404
		VIT_17s0000g02470	Thaumatin-like protein (VvPR-5)	1.963117801	0.026476649
		VIT_04s0023g00920	Endoribonuclease dicer 2 (VvDcr2)	1.871329189	0.000418918
		VIT_13s0019g01880	RN_N domain-containing protein	1.814450047	0.027100125
		VIT_00s0467g00030	RN_N domain-containing protein	1.758619774	0.040236181
		VIT_18s0001g03900	Toll/interleukin-1 receptor/resistance (TIR) domain-	1.564979972	0.003839959

		containing protein		
	VIT_13s0084g00420	RN_N domain-containing protein	1.548344919	0.02351503
	VIT_05s0077g01540	Pathogenesis-related protein 10.3 (VvPR-10.3)	1.478008918	4.26639E-07
	VIT_12s0034g00910	Toll/interleukin-1 receptor/resistance (TIR) domain-containing protein	1.457867985	0.002738712
	VIT_18s0041g02120	Toll/interleukin-1 receptor/resistance (TIR) domain-containing protein	1.397412817	0.010065127
	VIT_13s0019g04940	Protein RNA-directed DNA methylation 3 (VvRDM3)	1.346932252	0.003839959
	VIT_18s0089g00100	Toll/interleukin-1 receptor/resistance (TIR) domain-containing protein	1.302225064	0.012275989
	VIT_12s0034g01030	RN_N domain-containing protein	1.283562821	0.007584517
	VIT_18s0001g12390	Beta-1,3-glucanase (VvPR-2)	1.236363755	0.001666823
	VIT_11s0149g00120	Dicer-like protein 4 (VvDCL4)	1.16112013	0.038183156
	VIT_08s0040g00070	Protein argonaute 4A (VvAGO4A)	1.133509745	0.039592633
	VIT_06s0061g01040	Protein argonaute 5 (VvAGO5)	1.132398829	0.014819184

			VIT_12s0059g01320	Beta-1,3-glucanase (VvPR-2)	1.125450533	0.035729415
			VIT_04s0008g05430	RNA dependent RNA polymerase 6 (VvRdRp6)	1.105707065	0.008590499
			VIT_01s0011g05880	RNA dependent RNA polymerase 1 (VvRdRp1)	0.961610426	0.020025787
			VIT_18s0041g01750	NB-ARC domain-containing protein	0.837814871	0.039394598
	Berry	E-L 38	VIT_05s0077g01530	Pathogenesis-related protein 10.3 (VvPR-10.3)	1.864331635	0.000211093
			VIT_13s0019g01180	Beta-1,3-glucanase (VvPR-2)	1.039286395	6.09535E-05
Heat shock protein (HSP) and HSP co-chaperons	Leaf	E-L 31	VIT_04s0008g01570	17.3 kDa HSP class II	9.226044184	9.22E-05
			VIT_04s0008g01550	17.3 kDa HSP class II	7.603957955	0.012837145
			VIT_04s0008g01510	17.3 kDa HSP class II	6.424988963	0.002138398
			VIT_02s0025g00280	HSP90	3.025376811	0.002847964
			VIT_13s0019g02850	18.2kDa HSP class I	2.377512808	0.023444084
			VIT_13s0019g02740	18.2kDa HSP class I	2.125954671	0.008010291
			VIT_10s0003g00260	DNAJ/Hsp40 protein	1.314887474	0.040859804
			VIT_00s1569g00010	HSP90	1.208769929	0.026300316
			VIT_13s0019g01430	HSP70	1.185124836	0.010733371
			VIT_09s0070g00380	HSP90 activator	1.045716007	0.033708586
			VIT_13s0019g03170	18.2kDa HSP class I	0.957458782	0.000238353

		VIT_06s0080g01230	DNAJ/Hsp40 protein	0.841258972	0.011673479
Leaf	E-L 35	VIT_18s0001g14500	HSP90	3.156334541	2.72761E-05
		VIT_10s0003g04270	BAG family molecular chaperone regulator 3 (BAG3)	2.965573919	0.024144198
		VIT_09s0096g00090	HSP70	2.888006012	8.45249E-07
		VIT_11s0118g00490	HSP-interacting protein (HIP)	2.465038967	0.04367104
		VIT_07s0005g01220	DNAJ/Hsp40 protein	2.28557097	0.011353252
		VIT_00s1569g00010	HSP90	1.287425834	0.041107782
Berry	E-L 31	N/A	N/A	N/A	N/A
Berry	E-L 35	VIT_02s0025g00280	HSP83	1.158479187	0.007710556
		VIT_00s0415g00030	HSP70, mitochondrial	0.825741603	0.019977404
		VIT_01s0010g00680	HSP90-5, chloroplastic	0.750780825	0.046397019
Berry	E-L 38	VIT_16s0098g01060	HSP21	1.830294801	0.000308544
		VIT_06s0004g05770	17.3kDa class III	1.574898165	0.007365313
		VIT_13s0019g03000	18.1kDa class I	1.551780755	0.000917532
		VIT_18s0089g01270	22.0 kDa class IV	1.360046204	1.86909E-06
		VIT_18s0001g01610	17.3kDa class I	1.29956966	0.006848919
		VIT_18s0001g01570	17.3kDa class I	1.121770431	0.00045774
		VIT_11s0037g00510	HSP70	1.096414066	0.01360204
		VIT_04s0008g01610	17.3kDa class II	1.003144123	0.029437963

Transcription factors	Leaf	E-L 31	VIT_16s0013g01090	ERF005	5.063922151	0.030228975
			VIT_09s0018g00240	WRKY 40	2.092475714	0.014990795
			VIT_01s0026g01550	Homeobox-leucin zipper protein ATHB1	1.612778783	0.018890721
			VIT_01s0011g00720	WRKY 57	1.365749217	7.60E-05
			VIT_05s0077g00730	WRKY 48	1.139315463	0.041001274
			VIT_19s0014g01680	Transcription factor TCP4	1.127692628	1.35E-08
			VIT_07s0031g00080	WRKY 7	0.996325944	0.02961563
	Leaf	E-L 35	VIT_18s0001g03240	ERF004	8.244571753	0.037902476
			VIT_17s0000g01280	WRKY53	7.099495632	0.017285867
			VIT_14s0081g00670	AT-hook motif nuclear-localized protein 19 (AHL19)	6.267303956	0.009159622
			VIT_09s0002g09120	ERF003	4.662848083	0.042186627
			VIT_02s0012g01040	NAC domain-containing protein 86	4.52541628	5.07771E-10
			VIT_08s0007g07670	NAC transcription factor 47	4.106076888	0.001243178
			VIT_04s0008g06000	ERF045	3.704048387	0.009872403
			VIT_19s0090g00840	WRKY 31	2.820823282	0.020245924
			VIT_17s0000g03370	Protein SAR DEFICIENT 1 (SARD1)	2.584324183	0.026530846
			VIT_04s0008g05750	WRKY 18	2.402409344	0.01842931
			VIT_17s0000g03390	Protein SAR DEFICIENT 1 (SARD1)	2.314028131	0.021951318
			VIT_07s0031g01710	WRKY51	1.884566809	0.003513756

Berry	E-L 31	N/A	N/A	N/A	N/A
Berry	E-L 35	N/A	N/A	N/A	N/A
Berry	E-L 38	VIT_14s0068g01040	ERF113	2.307012083	0.002041952
		VIT_01s0011g03070	ERF RAV1	1.827804033	2.19481E-05
		VIT_19s0014g02240	ERF004	1.360965523	0.006348559
		VIT_18s0072g00260	ERF110	1.180480196	0.029758167
		VIT_12s0028g03270	ERF004	0.787263101	0.003857149
